# Phase 1b study of tirabrutinib in combination with idelalisib or entospletinib in previously treated B-cell lymphoma

**DOI:** 10.1038/s41375-020-01108-x

**Published:** 2020-12-17

**Authors:** Franck Morschhauser, Martin J. S. Dyer, Harriet S. Walter, Alexey V. Danilov, Loic Ysebaert, Daniel James Hodson, Christopher Fegan, Simon A. Rule, John Radford, Guillaume Cartron, Krimo Bouabdallah, Andrew John Davies, Stephen Spurgeon, Nishanthan Rajakumaraswamy, Biao Li, Rita Humeniuk, Xi Huang, Pankaj Bhargava, Juliane M. Jürgensmeier, Gilles Salles

**Affiliations:** 1 Univ. Lille, CHU Lille, ULR 7365, GRITA - Groupe de Recherche sur les formes Injectables et les Technologies Associées, F-59000 Lille, France; 2 Ernest and Helen Scott Haematological Research Institute, University of Leicester, Leicester, UK; 3 City of Hope National Medical Center, Duarte, CA USA; 4 Département d’Hématologie IUCT-Oncopole, Toulouse, France; 5 WT-MRC Stem Cell Institute, University of Cambridge, Cambridge, UK; 6 University Hospital of Wales, Cardiff, UK; 7 University of Plymouth Medical School, Plymouth, UK; 8 University of Manchester and The Christie NHS Foundation Trust, Manchester, UK; 9 Department of Clinical Hematology, University Hospital of Montpellier and UMR-CNRS 5535, Montpellier, France; 10 Hematology Clinic, University Hospital of Bordeaux, Pessac, France; 11 Cancer Research UK Centre, University of Southampton, Southampton, UK; 12 Knight Cancer Institute, Oregon Health and Science University, Portland, OR USA; 13 Gilead Sciences, Inc, Foster City, CA USA; 14 Hospices Civils de Lyon, Department of Hematology, Université de Lyon, Lyon, France

**Keywords:** Drug development, Cancer therapy

## To the Editor:

B-cell receptor (BCR) signaling pathway inhibitors (including Bruton’s tyrosine kinase [BTK] inhibitors, and phosphatidylinositol-3 kinase inhibitors [PI3Ki]) have shown clinical efficacy in non-Hodgkin lymphoma (NHL). However, responses to these agents have been limited in depth and duration. This may be due to resistance to PI3Kδ and BTK inhibitors as monotherapy [1–5]. The emergence of resistant clones may be addressed by combining these 2 classes of drugs. Furthermore, tolerability of these drug classes has been a concern. Combination therapy using lower doses of one or more classes of inhibitors may address some limitations.

Tirabrutinib (TIRA, formerly ONO/GS-4059) is a selective, irreversible, and small-molecule BTK inhibitor [6, 7]. TIRA has greater target selectivity compared with ibrutinib, which is characterized by high-affinity inhibition of ten kinases other than BTK [8]. In two separate phase 1 studies, TIRA was evaluated for treatment of patients with follicular lymphoma (FL), non-germinal center B-cell (non-GCB) diffuse large B-cell lymphoma (DLBCL), mantle cell lymphoma (MCL), and lymphoplasmacytic lymphoma (LPL) [7, 9]. Response rates were 20–100% across disease types, with a favorable safety profile [7, 9]. The only cardiovascular adverse events (AEs) were atrial fibrillation/flutter, which occurred in 5 of the 107 patients in the 2 studies, 4 of whom had this as a pre-existing medical condition.

We assessed the combination of TIRA with a PI3Kδ inhibitor (idelalisib [IDELA]) or a SYK inhibitor (entospletinib [ENTO]). IDELA is a first-in-class PI3Kδ inhibitor approved for the treatment of relapsed or refractory (R/R) chronic lymphocytic leukemia (CLL), small lymphocytic lymphoma (SLL), and FL in the US and Europe [10]. Response rates were 54% and 58% in FL and SLL, respectively [10]. ENTO (formerly GS-9973) is an investigational selective noncovalent inhibitor of SYK. In a study of patients with FL, LPL, MCL, and marginal zone lymphoma (MZL), treatment with ENTO 800 mg twice daily resulted in response rates ranging from 12% to 35%, with manageable toxicity [11].

The present study was designed to evaluate the safety of TIRA/IDELA and TIRA/ENTO combinations and define the maximum tolerated dose (MTD) for each combination and preliminary efficacy in patients with selected subtypes of NHL and CLL. The results from CLL patients have been reported elsewhere [12]. Herein, we report results in NHL patients.

This was a phase 1b, open-label, multicenter, sequential dose-escalation and -expansion study in patients with R/R NHL (NCT02457598). Eligible patients had a diagnosis of non-GCB DLBCL, FL, MCL, or other indolent NHL (MZL, SLL, or LPL) as documented by medical records. Patients had a history of ≥1 prior therapy (but no prior exposure to BTK or PI3K inhibitors), were not transplant eligible, and had either progressive disease (PD) or no response (stable disease) on their most recent treatment regimen. Except for those with LPL, patients had a radiologically measurable presence of ≥1 lymph node lesions.

Patients were treated with TIRA ranging from 20 mg to 160 mg QD in combination with IDELA (50 mg BID or 100 mg QD) or ENTO (200 mg QD or 400 mg QD) according to a standard 3 + 3 dose-escalation schema (Supplementary Table 1).

The primary endpoint was safety, evaluated by the occurrence of AEs, and laboratory abnormalities defined as dose-limiting toxicities (Supplementary Table 2). Preliminary efficacy was evaluated by overall response rate (ORR). Secondary endpoints included pharmacokinetic (PK) parameters, progression-free survival (PFS), duration of response, time to response, and proportion of patients achieving both complete response and undetectable minimal residual disease (MRD). Patients had CT or MRI scans every 12 weeks; those with DLBCL had an additional scan at week 6. Further details on study methods are provided in the Supplementary Methods.

Overall, 40 patients were treated with TIRA/IDELA and 91 with TIRA/ENTO (Supplementary Fig. 1). Patient characteristics and baseline demographics of the NHL subtypes included in this study are shown in Table 1. Forty-seven percent of DLBCL patients in the TIRA/IDELA group were refractory to their last prior regimen; 67% were refractory to prior regimens in the TIRA/ENTO group. In the TIRA/IDELA group, 70% of FL patients had failed ≥2 prior therapies, and 50% had failed both rituximab/anti-CD20+ and alkylating regimens. Eighty percent were refractory to their last prior treatment regimen before enrolling in this study, with stable disease or PD as the best prior response. In the TIRA/ENTO group, most patients were relapsed rather than refractory, with 65% of FL patients having achieved a response (27% complete, 38% partial) on their last prior therapy.

**Table 1:** Patient demographics and baseline characteristics.

	DLBCL *N* = 56^a^	FL *N* = 36	MCL *N* = 12	Other indolent NHL *N* = 27
*TIRA/IDELA, N*	17	10	1	12
Age, median (range) years	72 (37–89)	58 (32–70)	70 (70–70)	68 (41–77)
Female, *n* (%)	5 (29)	4 (40)	0	5 (42)
Time since diagnosis, median (range) years	1.5 (0.3–7.4)	9.3 (0.3–23.0)	3.3 (3.3–3.3)	6.0 (0.0–9.4)
Ann Arbor staging, *n* (%)^a^				
I–II	3 (18)	–	0	0
III or IV	14 (82)	–	1 (100)	11 (92)
Missing	0	–	0	1 (8)
Follicular lymphoma staging, *n* (%)				
Grade 1	–	0	–	–
Grade 2	–	7 (70)	–	–
Grade 3a	–	3 (30)	–	–
Unknown	–	0	–	–
ECOG performance status, *n* (%)				
0	9 (53)	3 (30)	0	7 (58)
1	8 (47)	7 (70)	1 (100)	2 (25)
≥2	0	0	0	2 (17)
Lactate dehydrogenase, U/L, median (range)	256 (157–924)	256 (176–970)	237 (237–237)	207 (72–411)
Prior no. of anticancer therapies, median (range)	3.0 (1–4)	4.0 (2–6)	2.0 (2–2)	2.5 (2–5)
Best response to last regimen, *n* (%)				
Complete response	2 (12)	0	0	2 (17)
Partial response	5 (29)	1 (10)	1 (100)	1 (8)
Stable disease	2 (12)	4 (40)	0	1 (8)
Progressive disease	6 (35)	4 (40)	0	4 (33)
Unknown	2 (12)	1 (10)	0	3 (25)
N/A	0	0	0	1 (8)
*TIRA/ENTO, N*	39	26	11	15
Age, median (range) years	69 (30–89)	67 (37–78)	70 (61–90)	68 (58–85)
Female	12 (31)	12 (46)	0	3 (20)
Time since diagnosis, median (range) years	1.7 (0.4–23.7)	6.1 (0.3–37.7)	4.6 (1.5–17.1)	6.1 (1.0–19.1)
Ann Arbor staging, *n* (%)^b^				
I–II	7 (18)	–	1 (9)	3 (20)
III or IV	32 (82)	–	9 (82)	10 (67)
Missing	0	–	1 (9)	2 (13)
Follicular lymphoma staging, *n* (%)				
Grade 1	–	3 (12)	–	–
Grade 2	–	12 (46)	–	–
Grade 3a	–	7 (27)	–	–
Unknown	–	4 (15)	–	–
ECOG performance status, *n* (%)				
0	19 (49)	17 (65)	5 (46)	10 (67)
1	18 (46)	8 (31)	6 (55)	5 (33)
≥2	2 (5)	1 (4)	0	0
Lactate dehydrogenase, median (range)	250 (131–956)	222 (131–492)	208 (146–408)	156 (107–1107)
Prior no. of anticancer therapies, median (range)	3.0 (1–7)	3.0 (1–12)	3.0 (2–4)	2.0 (1–6)
Best response to last regimen, *n* (%)				
Complete response	7 (18)	7 (27)	4 (36)	3 (20)
Partial response	6 (15)	10 (39)	2 (18)	5 (33)
Stable disease	12 (31)	2 (8)	3 (27)	4 (27)
Progressive disease	14 (36)	5 (19)	0	1 (7)
Unknown	0	2 (8)	1 (9)	2 (13)
N/A	0	0	1 (9)	0

^a^Fifty-five of the 56 DLBCL patients had non-GCB subtype, and 1 patient in the TIRA/IDELA group had GCB subtype (a protocol deviation resulting from a delayed pathology report).

^b^Follicular lymphoma grading was missing for 4 patients.

Study drug exposures are shown in Supplementary Fig. 2 and Supplementary Table 3. In the TIRA/IDELA and TIRA/ENTO groups, discontinuations due to AEs occurred in 10% and 3% of patients, and discontinuations due to PD occurred in 60% and 48% of patients, respectively. Five patients on TIRA/IDELA and 6 patients on TIRA/ENTO died on-study, all due to PD.

In the TIRA/IDELA treatment group, treatment-emergent AEs (TEAEs) occurred in 100% of patients, with 38% having serious TEAEs (Table 2). TEAEs leading to investigator-determined TIRA discontinuation occurred in 15% of TIRA/IDELA patients. These included anemia, neutropenia, pancytopenia, atrial fibrillation, and transaminase increased (1 patient each), and rash (*n* = 2). TEAEs leading to discontinuation of IDELA occurred in 18% of patients. Grade ≥3 laboratory abnormalities occurred in 69% of TIRA/IDELA patients. The most common hematologic and hepatic abnormalities (excluding lymphopenia) were decreased neutrophils and platelets, and increased liver enzymes (Supplementary Table 4). One MZL patient receiving TIRA 20 mg BID + IDELA 50 mg BID experienced a dose-limiting toxicity (grade 4 neutropenia and grade 4 thrombocytopenia), and subsequently the protocol was amended to include only TIRA QD dosing. MTD with TIRA QD dosing in combination with IDELA was not reached.

**Table 2:** Incidence of treatment-emergent adverse events.

TEAE summary	TIRA/IDELA *N* = 40		TIRA/ENTO *N* = 91	
TEAEs, *n* (%)	40 (100)		86 (95)	
TEAEs related to TIRA	31 (78)		70 (77)	
TEAEs leading to TIRA discontinuation	6 (15)		3 (3)	
TEAEs related to IDELA	31 (78)		–	
TEAEs leading to IDELA discontinuation	7 (18)		–	
TEAEs related to ENTO	–		64 (70)	
TEAEs leading to ENTO discontinuation	–		7 (8)	
SAEs^a^, *n* (%)	15 (38)		33 (36)	
SAEs related to TIRA	7 (18)		8 (9)	
SAEs related to IDELA	8 (20)		–	
SAEs related to ENTO	–		9 (10)	
**TEAEs by MedDRA preferred term**^b^	**TIRA/IDELA**		**TIRA/ENTO**	
	**Any grade**	**Grade** ≥**3**	**Any grade**	**Grade** ≥**3**
Neutropenia	11 (28)	9 (23)	14 (15)	14 (15)
Diarrhea	9 (23)	1 (3)	23 (25)	0 (0)
Thrombocytopenia	9 (23)	5 (13)	5 (6)	3 (3)
Pyrexia	8 (20)	1 (3)	13 (14)	4 (4)
Cough	8 (20)	0 (0)	10 (11)	0 (0)
Rash	8 (20)	1 (3)	9 (10)	1 (1)
Fatigue	6 (15)	0 (0)	20 (22)	0 (0)
Nausea	6 (15)	0 (0)	15 (17)	0 (0)
Back pain	6 (15)	1 (3)	12 (13)	0 (0)
Contusion	6 (15)	0 (0)	11 (12)	0 (0)
Headache	6 (15)	1 (3)	7 (8)	0 (0)
Vomiting	6 (15)	1 (3)	9 (10)	0 (0)
Decreased appetite	6 (15)	0 (0)	7 (8)	0 (0)
Dyspnea	6 (15)	2 (5)	10 (11)	1 (1)
Rash, maculopapular	6 (15)	4 (10)	2 (2)	1 (1)
Asthenia	2 (5)	0 (0)	21 (23)	1 (1)

*MedDRA* Medical Dictionary for Regulatory Activities.

^a^SAEs occurring in ≥2 patients in either treatment group.

^b^TEAEs are shown according to incidence of any grade event occurring in ≥15% of patients in either treatment group.

In the TIRA/ENTO treatment group, TEAEs occurred in 95% of patients, with 36% having serious TEAEs (Table 2). TEAEs leading to investigator-determined TIRA discontinuation occurred in 3% of TIRA/ENTO patients. These included biphasic mesothelioma, lung neoplasm (malignant), and pancreatitis (one patient each). TEAEs leading to discontinuation of ENTO occurred in 8% of patients. Grade ≥3 laboratory abnormalities occurred in 57% of TIRA/ENTO patients. The most common hematologic and hepatic abnormalities (excluding lymphopenia) were decreased neutrophils and platelets, and increased liver enzymes (Supplementary Table 4). There were no dose-limiting toxicities in the TIRA/ENTO group and MTD was not reached.

Efficacy results are reported across all dose groups in both combinations. Response rates are shown in Supplementary Table 5. In DLBCL patients, response rates were comparable across both combinations; the ORR was 24% with TIRA/IDELA and 26% with TIRA/ENTO. The ORR in patients with FL was 20% with TIRA/IDELA and 35% with TIRA/ENTO, and in patients with MCL, ORR was 100% with TIRA/IDELA and 64% with TIRA/ENTO. In patients with other indolent NHL, the ORR was 58% with TIRA/IDELA and 67% with TIRA/ENTO. The median (Q1, Q3) time to response and the median (Q1, Q3) duration of response with TIRA/IDELA and TIRA/ENTO for DLBCL, FL, MCL, and other indolent NHL are shown in Supplementary Table 5; Kaplan–Meier estimated duration of response is shown separately for each NHL subgroup in Supplementary Table 6. A ≥50% sum of the products of the longest perpendicular diameters (SPD) reduction from baseline occurred in 29% and 26% of DLBCL patients, in 20% and 35% of FL patients, and in 38% and 42% of patients with other NHL subtypes (including MCL) on TIRA/IDELA and TIRA/ENTO, respectively (Supplementary Fig. 3). PFS is shown in Supplementary Fig. 4 and MRD status in Supplementary Fig. 5.

The primary objective of this phase 1 study was to assess the safety and tolerability of the TIRA/IDELA and TIRA/ENTO combinations. The AE rates were generally comparable to those observed in previous studies of TIRA [7, 9], IDELA [13], or ENTO [14] monotherapy in patients with NHL. There were no unanticipated safety signals seen with either treatment combination at all doses, and the MTD for either combination was not reached. IDELA-associated toxicities appeared to be less frequent in our study compared to what has been reported previously, possibly due to the use of lower doses [10]. In particular, no patients receiving TIRA/IDELA had serious TEAEs of diarrhea, colitis, or ALT/AST elevation.

The combination of TIRA + low-dose IDELA induced responses in only 20% of FL patients, and the combination of TIRA + ENTO yielded a 35% response rate. These ORRs are lower than those observed in other studies of R/R FL, including treatment with the approved dose of 150 mg twice daily IDELA (54% ORR) [2, 10], copanlisib (40–55% ORR) [3, 15], and duvelisib (42% ORR) [4]. The suboptimal responses in the present study may be due to lower IDELA dosing. Except in the DLBCL cohort, where the 160 mg TIRA QD dose increased IDELA plasma PK exposure relative to lower TIRA doses due to drug-drug interaction, no further impact of TIRA on IDELA PK at lower doses, or additional drug-drug interactions were observed in the study [12]. Consistent with a previous IDELA dose-ranging study [13], outcomes in DLBCL patients treated with TIRA/IDELA (*N* = 17) suggest that reduced IDELA exposure had a detrimental impact on efficacy.

The overall hypothesis for this study was to explore whether combining different BCR-targeted agents at lower doses than their respective doses in monotherapy offers a meaningful strategy to increase tolerability while preserving or improving efficacy. Based on the results of this study, we conclude that while the explored combinations of TIRA/IDELA and TIRA/ENTO were overall well tolerated, there was no meaningful efficacy advantage to be gained with this approach. It is possible that any advantage achieved in safety through lower doses may in fact have compromised efficacy in this study.

## Supplementary information

Phase 1b Study of Tirabrutinib in Combination With Idelalisib or Entospletinib in Previously Treated B-Cell Lymphoma Supplemental Material Franck Morschhauser, et al.
