# Supplementary material for: Phase 1b study of tirabrutinib in combination with idelalisib or entospletinib in previously treated B-cell lymphoma
Source: Leukemia. 2020 Dec 17;35(7):2108–13. doi: 10.1038/s41375-020-01108-x (PMC8257485; doi:10.1038/s41375-020-01108-x)
Supplement: Supplementary file 1 — Phase 1b Study of Tirabrutinib in Combination With Idelalisib or Entospletinib in Previously Treated B-Cell Lymphoma Supplemental Material Franck Morschhauser, et al. [file 41375_2020_1108_MOESM1_ESM.pdf]

Phase 1b Study of Tirabrutinib in Combination With Idelalisib or Entospletinib in Previously Treated B-Cell Lymphoma

Supplemental Material

Franck Morschhauser, et al.

Supplemental Methods

Patients and treatment

Eligible patients had a diagnosis of non-germinal center B-cell (GCB) diffuse large B-cell lymphoma (DLBCL), follicular lymphoma (FL), mantle cell lymphoma (MCL), or other indolent non-Hodgkin lymphoma (NHL) (marginal zone lymphoma [MZL], small lymphocytic lymphoma [SLL], or lymphoplasmacytic lymphoma [LPL]) as documented by medical records and with histology based on the World Health Organization criteria that were current at the time of patient enrollment.[1-3] DLBCL subtyping was performed locally according to the Hans classification algorithm.[4] FL was limited to histological grades 1, 2, and 3a. MZL could be splenic, nodal, or extranodal. Patients with SLL had an absolute lymphocyte count of $<5 \times 10^9$/L at initial diagnosis, and those with LPL had measurable disease defined as serum monoclonal immunoglobulin M > 0.5 g/dL or meeting at least 1 of the recommendations from the Second International Workshop on Waldenström's Macroglobulinemia for requiring treatment. No central independent histopathological review was performed.

The disease and dose chosen for expansion cohorts were based on emerging safety, efficacy, pharmacokinetic (PK), and pharmacodynamic results of the dose-escalation phase. Based on the safety, PK and biomarker data from a previous study[5], the initial dose regimens were tirabrutinib (TIRA) 20 mg QD + idelalisib (IDELA) 50 mg BID, and TIRA 40 mg QD + entospletinib (ENTO) 200 mg QD.

Endpoint definitions and analysis methods

Analysis results are presented using descriptive statistics. For categorical variables, the number and percentage of subjects in each category are presented; for continuous variables, this may include the number of subjects (n), median, first quartile (Q1), third quartile (Q3), minimum, and maximum. Overall response rate (ORR) was defined as the proportion of patients who achieve a complete response (CR) or partial response (PR) based on standardized response criteria for malignant lymphomas.[6-8] Best overall response (BOR) was defined as the best response recorded during the follow-up period after first dose prior to the time of initiation of anticancer therapy other than the study treatment. ORR is defined as the proportion of subjects who achieve a BOR of CR or PR during the study, except for patients with LPL for whom ORR includes CR, PR, very good PR, and minor PR. Progression-free survival (PFS) was analyzed by Kaplan-Meier methods, and was defined as the interval from the start of the study therapy to the earlier of the first documentation of definite radiographic disease progression or death from any cause.

Pharmacokinetic assessments

PK assessments were conducted as previously described.[9] Patients in the dose-escalation cohorts underwent intensive PK sampling on day 1, day 2, and day 8 of the first treatment cycle, and sparse PK sampling up to

Cycle 6 of treatment. TIRA and IDELA plasma concentrations were determined using validated bioanalytical assays. PK parameters were estimated by standard noncompartmental methods using Phoenix WinNonlin® 7.0 software (Certara, Princeton, NJ, US). PK concentrations and parameters were summarized using descriptive statistics.

Exploratory biomarker analyses

When available, archival tumor tissue was collected from DLBCL patients. Minimal residual disease (MRD) analysis for DLBCL patients was performed during cycle 1 and at disease progression/end of treatment by Adaptive Biotechnologies (Seattle, WA, US). Briefly, pretreatment FFPE tissue was analyzed to identify tumor-specific clonotypes on the basis of the ClonoSeq method.[10] Circulating tumor DNA (ctDNA) from serial plasma samples was then used for MRD surveillance on treatment. MRD positivity was defined as the level of tracked clonotypes exceeding detection limit, which is sample-specific and determined based on the BCR repertoire from the sample.

Supplemental Results

Pharmacokinetics

The pharmacokinetics of TIRA in patients with NHL were consistent with previous studies.[17, 25] Mean (% CV) TIRA peak plasma concentrations at 1.5-4 hours post-dose were 268 (27) and 381 (41) ng/mL in DLBCL patients receiving 80 mg (n=4) and 160 mg (n=3) TIRA QD (in combination with 100 mg IDELA QD). At the 80 mg TIRA + 100 mg IDELA QD dose level, peak TIRA plasma concentrations in other NHL (n=8) patients were 268 (34) ng/mL.

Coadministration of TIRA with IDELA did not affect TIRA PK; however, TIRA increased IDELA exposures at a higher dose (160 mg QD TIRA) that was evaluated in DLBCL patients.[25] IDELA exposures (AUCtau, mean [% CV]) were 9490 (34.4) and 19,300 (29.1) h*ng/mL in DLBCL patients assigned to 100 mg QD IDELA, in combination with 80 mg QD TIRA and 160 mg TIRA, respectively. The daily IDELA exposures (mean [%CV]) observed in this study were within the range of those previously established to be safe in patients with CLL and FL (150 mg BID IDELA; Cmax = 1861 [43%], AUCtau = 10598 [41%].[2]

ENTO PK was comparable at the 80 mg TIRA QD and 160 mg TIRA QD doses. In 3 patients receiving 80 mg TIRA QD + 400 mg ENTO QD, mean (% CV) peak ENTO plasma concentration on cycle 1 day 8 was 1249 (42%) ng/mL, compared with 827 (37%) ng/mL in 4 patients receiving 160 mg TIRA QD + 400 mg ENTO.

Biomarkers

Exploratory biomarker analyses were performed for the DLBCL cohort. After identifying patient-specific B-cell clones from archival tumor tissue, clone tracking from ctDNA was successful in 8 patients in the TIRA/IDELA group and in 22 patients on TIRA/ENTO (**Supplemental Figure 5**). In the TIRA/IDELA group, all patients with PD and SD had MRD detectable at all time points. Two patients with PR temporarily had no detectable MRD but did not maintain this status, while 1 patient with PR had no detectable ctDNA at baseline and remained MRD negative for 15 cycles (and is still on treatment). In the TIRA/ENTO group, 1 patient had no detectable

ctDNA at baseline, but became MRD positive at end of treatment. The only patient with a CR who had ctDNA tracking turned MRD negative on treatment at the first measured time point and remained MRD negative until end of treatment.

References

1. WHO classification of tumours of haematopoietic and lymphoid tissues. In: Bosman FT, Jaffe ES, Lakhani SR, Ohgaki H, eds. World Health Organization Classification of Tumours. IARC: Lyon, France, 2008.

2. Campo E, Swerdlow SH, Harris NL, Pileri S, Stein H, Jaffe ES. The 2008 WHO classification of lymphoid neoplasms and beyond: evolving concepts and practical applications. *Blood* 2011; **117:** 5019-5032.

3. Swerdlow SH, Campo E, Pileri SA, Harris NL, Stein H, Siebert R, *et al.* The 2016 revision of the World Health Organization classification of lymphoid neoplasms. *Blood* 2016; **127:** 2375-2390.

4. Hans CP, Weisenburger DD, Greiner TC, Gascoyne RD, Delabie J, Ott G, *et al.* Confirmation of the molecular classification of diffuse large B-cell lymphoma by immunohistochemistry using a tissue microarray. *Blood* 2004; **103:** 275-282.

5. Walter HS, Rule SA, Dyer MJ, Karlin L, Jones C, Cazin B, *et al.* A phase 1 clinical trial of the selective BTK inhibitor ONO/GS-4059 in relapsed and refractory mature B-cell malignancies. *Blood* 2016; **127:** 411-419.

6. Cheson BD, Pfistner B, Juweid ME, Gascoyne RD, Specht L, Horning SJ, *et al.* Revised response criteria for malignant lymphoma. *J Clin Oncol* 2007; **25:** 579-586.

7. Owen RG, Kyle RA, Stone MJ, Rawstron AC, Leblond V, Merlini G, *et al.* Response assessment in Waldenstrom macroglobulinaemia: update from the VIth International Workshop. *Br J Haematol* 2013; **160:** 171-176.

8. Hallek M, Cheson BD, Catovsky D, Caligaris-Cappio F, Dighiero G, Dohner H, et al. iwCLL guidelines for diagnosis, indications for treatment, response assessment, and supportive management of CLL. *Blood* 2018; **131:** 2745-2760.

9. Danilov AV, Herbaux C, Walter HS, Hillmen P, Rule SA, Kio EA, et al. Phase Ib study of tirabrutinib in combination with idelalisib or entospletinib in previously treated chronic lymphocytic leukemia. *Clin Cancer Res* 2020; **26:** 2810-2818.

10. Roschewski M, Dunleavy K, Pittaluga S, Moorhead M, Pepin F, Kong K, et al. Circulating tumour DNA and CT monitoring in patients with untreated diffuse large B-cell lymphoma: a correlative biomarker study. *Lancet Oncol* 2015; **16:** 541-549.

Supplemental Table 1. Dose-escalation cohorts (3+3 design).

	Group A		Group B[a]	
**Dose Level**	**TIRA**	**IDELA**	**TIRA**	**IDELA**
1[b]	20 mg QD	50 mg BID	—	—
2	40 mg QD	50 mg BID	20 mg BID	50 mg BID
3	80 mg QD	50 mg BID	—	—
4	80 mg QD	100 mg QD	—	—
5[c]	160 mg QD	100 mg QD	—	—
**Dose Level**	**TIRA**	**ENTO**	**TIRA**	**ENTO**
1	40 mg QD	200 mg QD	—	—
2[a]	80 mg QD	200 mg QD	40 mg QD	400 mg QD
3	150 mg QD	200 mg QD	80 mg QD	400 mg QD
4[c]	—	—	160 mg QD	400 mg QD

[a]TIRA/IDELA group B was closed to further enrollment following dose-limiting toxicity of neutropenia.
[b]Initially, 3 patients were enrolled in dose level 1A. The first cycle consisted of 28 days (1 day single agent TIRA and 27 days of combination treatment), and each subsequent cycle consisted of 28 days of combination treatment. If only 1 dose-limiting toxicity (DLT) occurred within 28 days from cycle 1, day 1, dose level 1A was expanded to enroll 3 additional patients. Subsequently if ≥2 patients experienced DLT, development of the combination of TIRA and IDELA was to be discontinued. If no DLT occurred in 3 patients or <2 DLTs occurred in up to 6 patients in Cohort 1A, then Cohort 2A was initiated. For the remainder of dose escalation, higher cohorts began enrollment if no DLTs in 3 patients or <2 DLTs in up to 6 patients were observed. If a second DLT was observed in any cohort, the maximum tolerated dose (MTD) of TIRA combined with IDELA or ENTO was considered to have been exceeded, and the prior dose level was considered to be the MTD. The MTD for TIRA once-daily was assessed separately from the MTD for TIRA twice daily. Once all 3 treatment cohorts were open, the choice of enrolling a patient into a cohort and dosing level was based on the treatment slots open at the time of screening and at the discretion of the investigator.
[c]This dose level was only administered to DLBCL patients.

Supplemental Table 2. Definition of dose-limiting toxicities[a]

**Grade ≥4**	• All grade ≥4 hematological toxicities persisting for >7 days.<br>• All grade ≥4 nonhematologic laboratory abnormalities.
**Grade ≥3**	All grade ≥3 nonhematological toxicities except for tumor lysis or alopecia, or grade 3 nausea, vomiting, diarrhea, or constipation that resolves within 72 hours with medical intervention.
**Grade ≥2**	Grade ≥2 nonhematologic TEAE that in the opinion of the investigator is of potential clinical significance such that further dose escalation would expose subjects to unacceptable risk.
**Febrile Neutropenia**	Febrile neutropenia defined as ANC <1.0 x 10^9/L with a single temperature >38.3°C (101°F) or sustained temperature ≥38°C (100.4°F) for more than 1 hour.

[a]For toxicities occurring between day 1 and day 28 of a dosing cycle.

Supplemental Table 3. Drug exposure.

	TIRA/IDELA				TIRA/ENTO			
	**DLBCL N=17**	**FL N=10**	**MCL N=1**	**Other Indolent NHL* N=12**	**DLBCL N=39**	**FL N=26**	**MCL N=11**	**Other Indolent NHL* N=15**
Duration of exposure to TIRA, median (range) weeks	7.7 (0.6, 61.1)	24.9 (2.1, 96.0)	88.6	80.3 (3.3, 166.4)	8.0 (1.3, 102.7)	27.7 (4.7, 103.0)	53.1 (0.1, 110.4)	76.4 (28.1, 166.4)
Dose interruption, n (%) patients	7 (41)	7 (70)	1 (100)	11 (92)	10 (25.6)	9 (34.6)	4 (36)	7 (47)
Adverse event	7 (41)	7 (70)	1 (100)	8 (67)	6 (15.4)	6 (23.1)	4 (36)	5 (33)
Noncompliance	0	0	0	2 (17)	2 (5.1)	0	0	0
Other	0	0	0	1 (8)	2 (5.1)	3 (11.5)	0	2 (13)
Dose modification, n (%) patients	3 (18)	1 (10)	1 (100)	4 (33)	2 (5.1)	1 (3.8)	3 (27)	2 (13)
Adverse event	0	0	1 (100)	0	0	0	0	0
Per protocol	3 (18)	1 (10)	0	1 (8)	2 (5.1)	1 (3.8)	2 (18)	1 (7)
Noncompliance	0	0	0	1 (8)	0	0	0	0
Other	0	0	0	2 (17)	0	0	1 (9)	1 (7)
Duration of exposure to IDELA/ ENTO, median (range) weeks	6.7 (0.4, 61.0)	18.1 (1.4, 95.9)	20.7	28 (3.1, 121.0)	7.9 (1.1, 85.7)	27.5 (4.6, 102.9)	46.4 (7.9, 110.3)	76.3 (28.0, 166.3)
Dose interruption, n (%) patients	7 (41)	6 (60)	0	9 (75)	10 (25.6)	8 (30.8)	3 (27)	6 (40)
Adverse event	7 (41)	6 (60)	0	7 (58)	6 (15.4)	5 (19.2)	3 (27)	4 (27)
Noncompliance	0	0	0	1 (8)	2 (5.1)	0	0	0
Other	0	0	0	1 (8)	2 (5.1)	3 (11.5)	0	2 (13)
Dose modification, n (%) patients	2 (12)	0	0	3 (25)	3 (7.7)	1 (3.8)	2 (18)	1 (7)
Adverse event	0	0	0	0	1 (2.6)	0	0	0
Per protocol	2 (12)	0	0	1 (8)	2 (5.1)	1 (3.8)	2 (18)	1 (7)
Noncompliance	0	0	0	1 (8)	0	0	0	0
Other	0	0	0	1 (8)	0	0	0	0

*LPL, MZL, SLL.

Supplemental Table 4. Laboratory abnormalities of interest.*

Laboratory abnormality, n (%)	**TIRA/IDELA N=40**		**TIRA/ENTO N=91**	
	**Any grade**	**Grade ≥3**	**Any grade**	**Grade ≥3**
Any laboratory abnormality	39 (100)*	27 (69)	88 (98)	51 (57)
Chemistry				
Albumin decreased	2 (5)	0	7 (8)	0
Alkaline phosphatase increased	10 (26)	1 (3)	11 (12)	0
Alanine transaminase increased	17 (44)	7 (18)	29 (32)	7 (8)
Amylase increased	1 (3)	0	4 (4)	1 (1)
Aspartate transaminase increased	17 (44)	2 (5)	19 (21)	5 (6)
Creatinine clearance decreased	13 (33)	0	42 (47)	0
Creatinine increased	1 (3)	0	13 (14)	0
γ-glutamyl transferase increased	12 (31)	3 (8)	21 (23)	2 (2)
Hyperbilirubinemia	1 (3)	0	13 (14)	1 (1)
Hypercalcemia (corrected calcium)	3 (8)	0	9 (10)	1 (1)
Hypercholesterolemia	3 (8)	0	3 (3)	0
Hyperglycemia	11 (28)	2 (5)	21 (23)	5 (6)
Hyperkalemia	0	0	1 (1)	0
Hypermagnesemia	2 (5)	0	3 (3)	0
Hypernatremia	2 (5)	0	6 (7)	0
Hyperuricemia	5 (13)	0	32 (36)	12 (13)
Hypocalcemia (corrected calcium)	2 (5)	0	0	0
Hypoglycemia	7 (18)	0	19 (21)	0
Hypokalemia	8 (21)	1 (3)	5 (6)	0
Hypomagnesemia	2 (5)	0	3 (3)	0
Hyponatremia	3 (8)	0	23 (26)	5 (6)
Hypophosphatemia	2 (5)	1 (3)	18 (20)	6 (7)
Lipase increased	2 (5)	0	6 (7)	1 (1)
Triglycerides increased	27 (69)	2 (5)	44 (49)	2 (2)
Coagulation				
Activated partial thromboplastin time	5 (13)	0	2 (2)	1 (1)
Prothrombin international normalized ratio	1 (3)	1 (3)	2 (2)	0
Hematology				
Hemoglobin decreased	14 (36)	2 (5)	24 (27)	1 (1)
Lymphocytes decreased	17 (44)	8 (21)	27 (30)	14 (16)
Lymphocytes increased	5 (13)	0	10 (11)	2 (2)
Neutrophils decreased	16 (41)	8 (21)	26 (29)	14 (16)
Platelets decreased	16 (41)	4 (10)	28 (31)	4 (4)
White blood cell count decreased	14 (36)	4 (10)	24 (27)	7 (8)
White blood cell count increased	2 (5)	2 (5)	1 (1)	1 (1)
Urinalysis				
Proteinuria	7 (19)	0	19 (22)	0

*Percentages are calculated based on number of patients with post-baseline values.

Supplemental Table 5. Overall response.

	DLBCL N=56	FL N=36	MCL N=12	Other Indolent NHL[‡] N=27
TIRA/IDELA, N	17	10	1	12
ORR*, n (%)<br>95% CI	4 (24)<br>7, 50	2 (20)<br>3, 56	1 (100)<br>2.5, 100	7 (58)<br>28, 85
Time to response,<br>median (Q1, Q3) months	1.7<br>1.5, 3.2	4.1<br>2.6, 5.5	10.8<br>--	2.8<br>2.7, 2.9
Duration of response, KM-estimated<br>median (Q1, Q3) months	6.4<br>3.9, NR	8.4<br>3.0, 13.8	10.7<br>--	19.3<br>3.3, NR
Best overall response, n (%)				
Complete response	1 (6)	0	0	0
Very good partial response (LPL)	--	--	--	0
Partial response	3 (18)	2 (20)	1 (100)	7 (58)
Minor response (LPL)	--	--	--	0
Stable disease	4 (24)	6 (60)	0	2 (17)
Progressive disease	7 (41)	1 (10)	0	1 (8)
Nonevaluable	0	0	0	0
Discontinued study[†]	2 (12)	1 (10)	0	2 (17)
TIRA/ENTO, N	39	26	11	15
ORR*, n (%)<br>95% CI	10 (26)<br>13, 42	9 (35)<br>17, 56	7 (64)<br>31, 89	10 (67)<br>38, 88
Time to response,<br>median (Q1, Q3) months	1.5<br>1.4, 2.8	2.8<br>2.8, 4.6	2.8<br>2.8, 2.8	4.1<br>2.8, 5.5
Duration of response, KM-estimated<br>median (Q1, Q3) months	NR<br>3.4, NR	16.6<br>11.5, 17.3	15.3<br>14.0, 16.6	NR<br>NR, NR
Best overall response, n (%)				
Complete response	4 (10)	2 (8)	3 (27)	2 (13)
Very good partial response (LPL)	--	--	--	0
Partial response	6 (15)	7 (27)	4 (36)	8 (53)
Minor response (LPL)	--	--	--	0
Stable disease	11 (28)	10 (39)	3 (27)	5 (33)
Progressive disease	13 (33)	5 (19)	0	0
Nonevaluable	1 (3)	0	0	0
Discontinued study[†]	4 (10)	2 (8)	1 (9)	0

*Overall response rate = complete response + partial response for all subtypes except Waldenström's macroglobulinemia, where overall response rate also includes very good partial response and minor response.
[†]Discontinued study or started new anticancer therapy before first assessment.
[‡]LPL, MZL, SLL.
KM, Kaplan-Meier; NR, not reached.

Supplemental Table 6. Kaplan-Meier estimated duration of response.

	TIRA/IDELA<br>N=40	TIRA/ENTO<br>N=91
Diffuse large B-cell lymphoma	6.4 (3.9, NR)	NR
Follicular lymphoma	8.4 (3.0, 13.8)	16.6 (11.5, 17.3)
Mantle cell lymphoma	10.7*	15.3 (14.0, 16.6)
Marginal zone lymphoma	2.9 (2.5, 3.3)	NR
Small lymphocytic lymphoma	19.3*	NR
Waldenström's macroglobulinemia/lymphoplasmacytic lymphoma	NR	NR

Data show median (Q1, Q3) duration of response in months.

*N=1 MCL and N=2 SLL patients in the TIRA/IDELA group.
NR, not reached.

Supplemental Figure 1. Patient disposition.

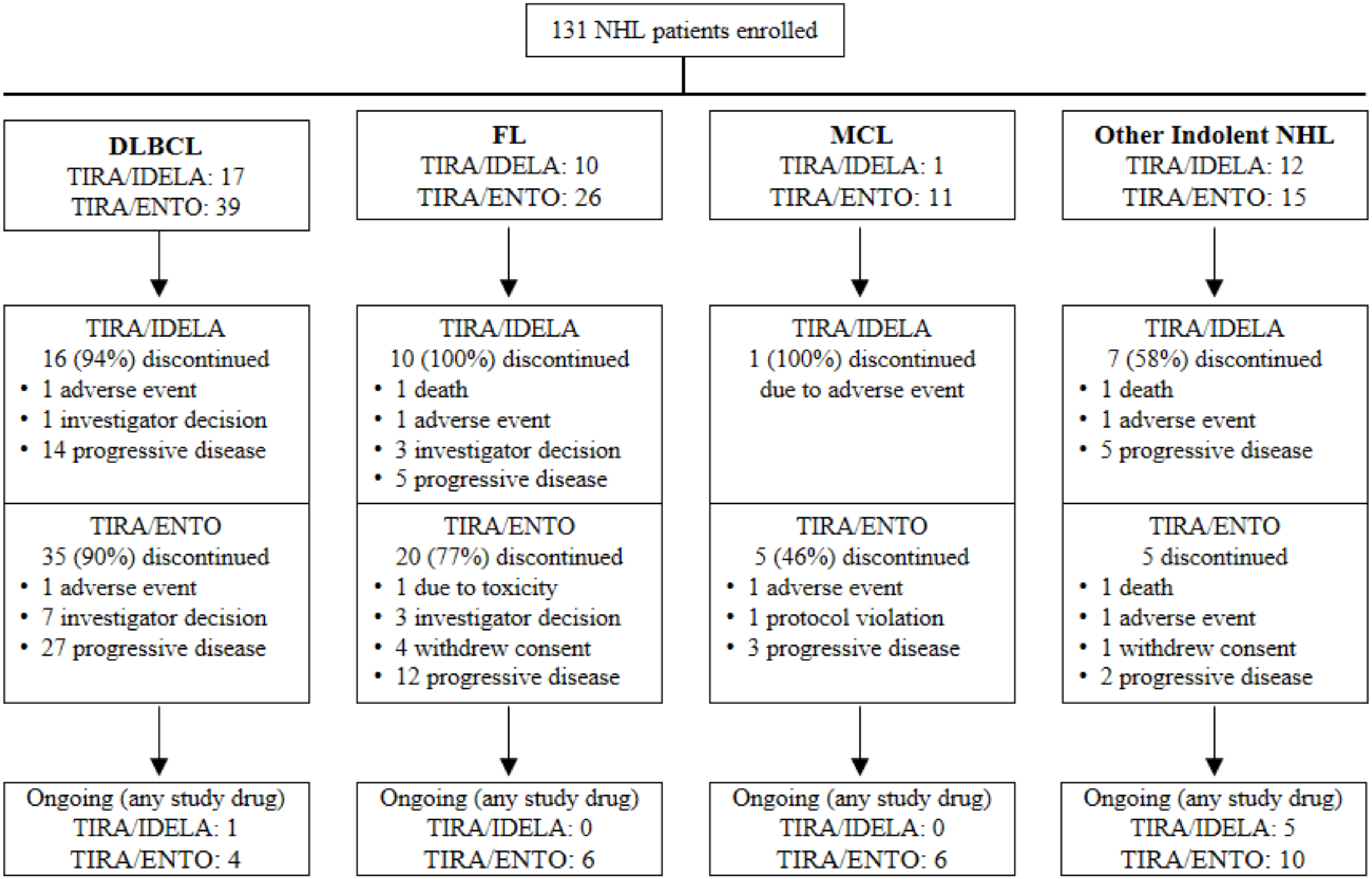

Supplemental Figure 2. Patient exposure and disposition in patients with DLBCL, FL, and Other NHL.

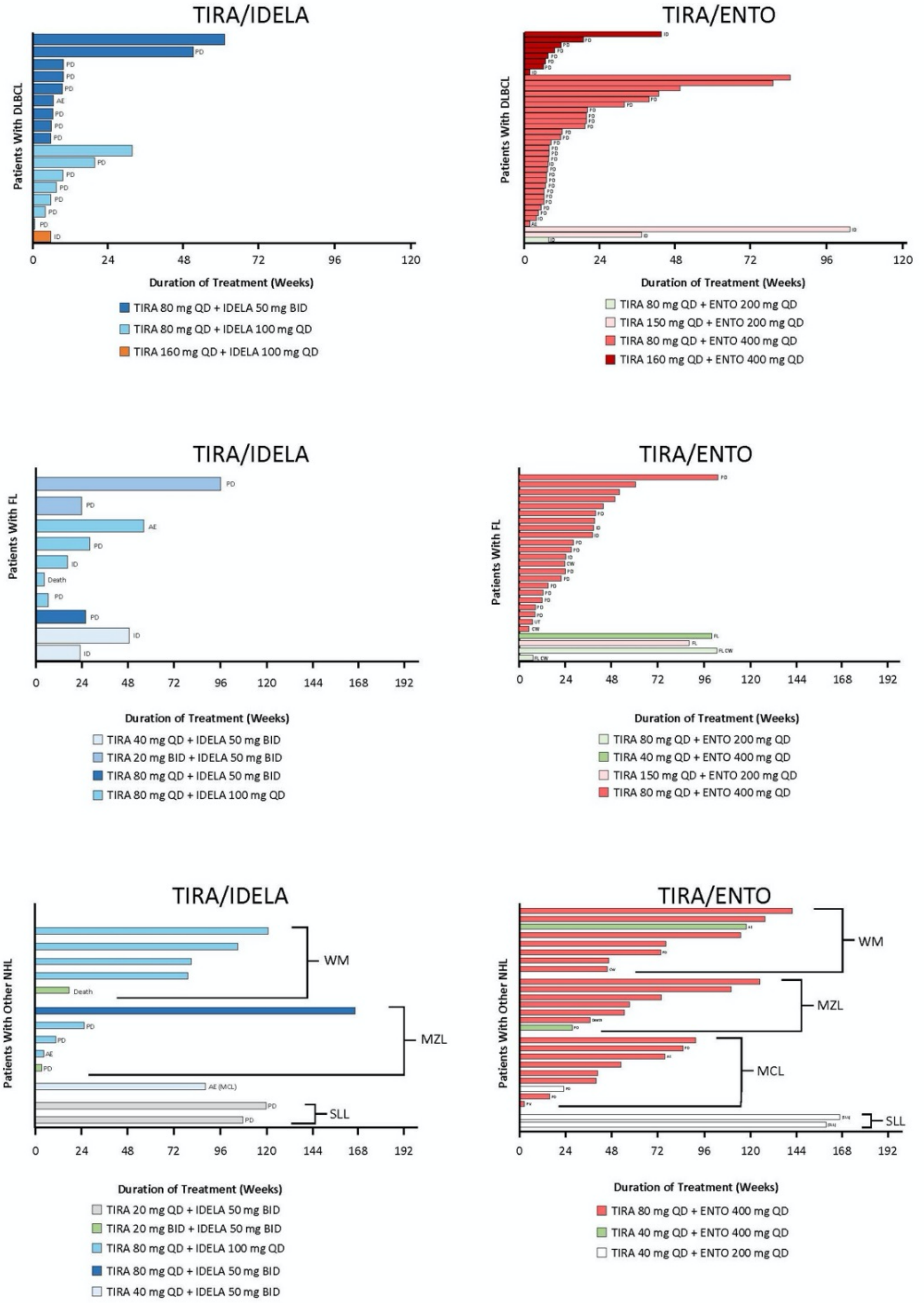

Supplemental Figure 3. Best SPD change from baseline in patients with A) non-GCB DLBCL, B) FL, and C) Other NHL.

A. IDELA: Patients with missing values (n=2) are not shown; ENTO: Patients with missing values (n=5) are not shown
B. IDELA: Patients with missing values (n=2) are not shown; ENTO: Patients with missing values (n=3) are not shown
C. IDELA: Patients with missing values (n=4) are not shown; ENTO: Patients with missing values (n=5) are not shown

Other NHL = SLL, MCL, MZL, LPL; SPD, sum of the products of greatest perpendicular diameters.

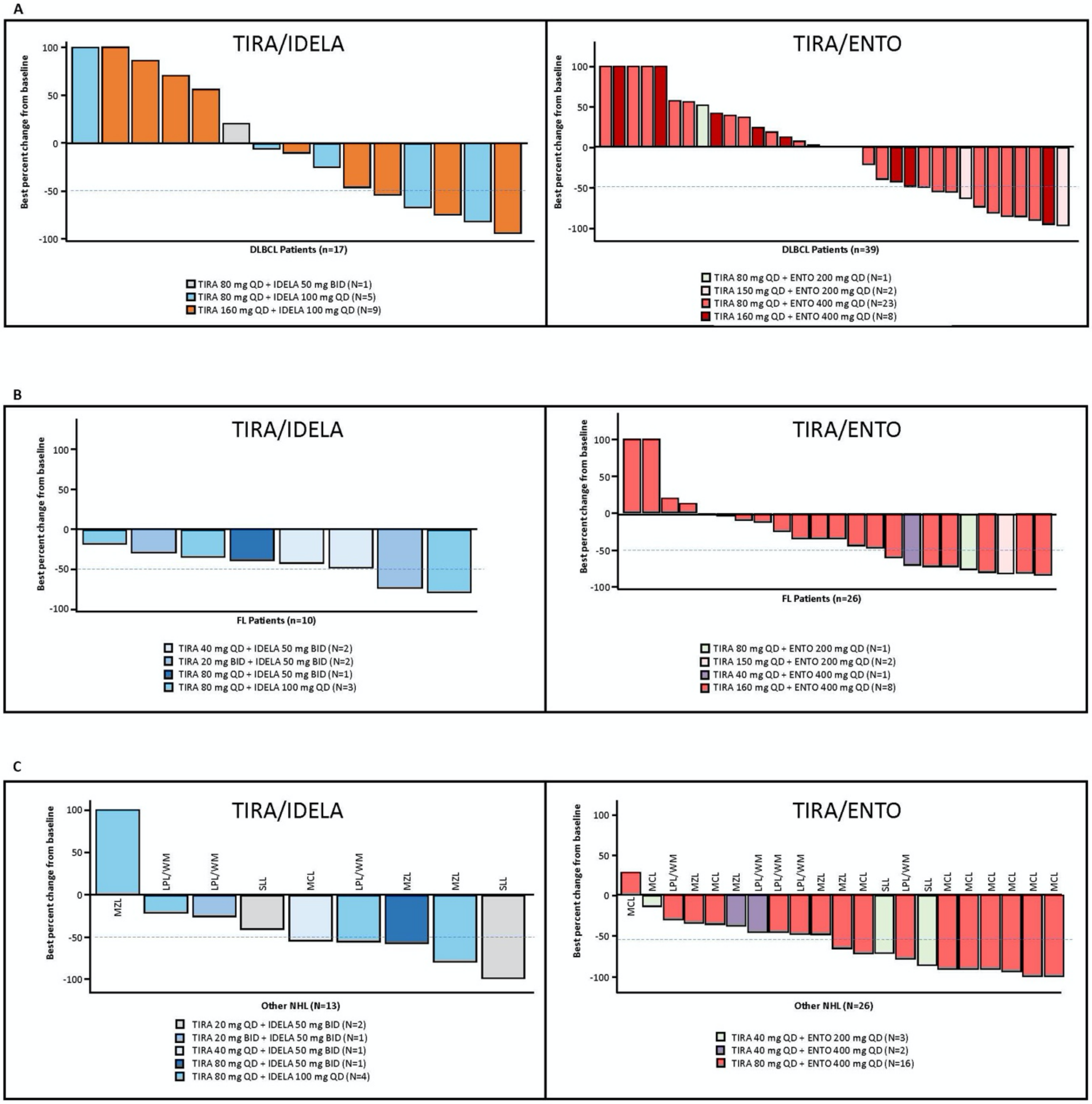

Supplemental Figure 4. Kaplan-Meier curve of PFS in patients treated with A) TIRA/IDELA and B) TIRA/ENTO.

A

	DLBCL	FL	MCL	Other NHL
Median PFS, months (Q1, Q3)	2.3 (1.4, 5.0)	5.4 (5.3, 11.1)	21.6 (21.6, 21.6)	6.0 (4.2, 21.9)

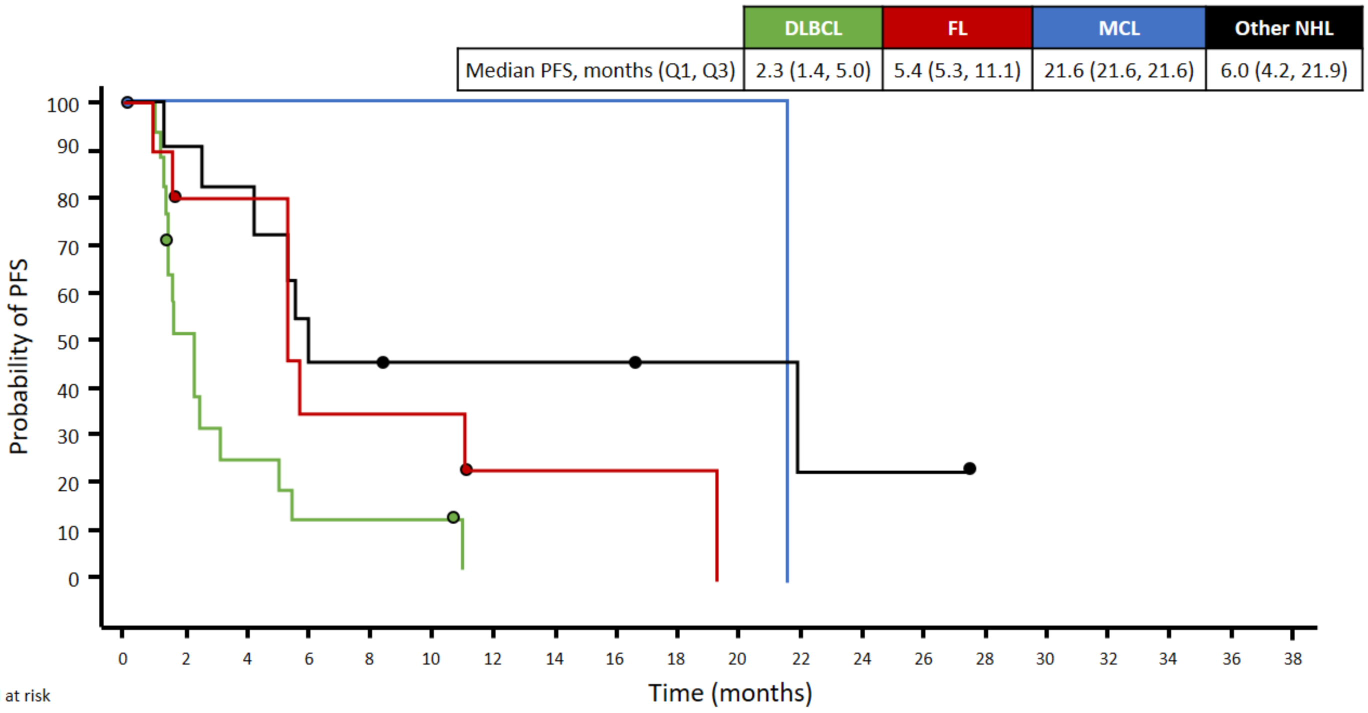

N at risk

	0	2	4	6	8	10	12	14	16	18	20	22	24	26	28
DLBCL	17	8	4	2	2	2	0								
FL	10	7	7	3	3	3	1	1	1	1	0				
MCL	1	1	1	1	1	1	1	1	1	1	1	0			
Other NHL	12	10	9	6	5	4	4	4	4	2	2	1	1	1	0

B

	DLBCL	FL	MCL	Other NHL
Median PFS, months (Q1, Q3)	2.8 (1.5, 8.2)	19.4 (5.6, 19.4)	19.4 (16.8, NR)	NR (NR, NR)

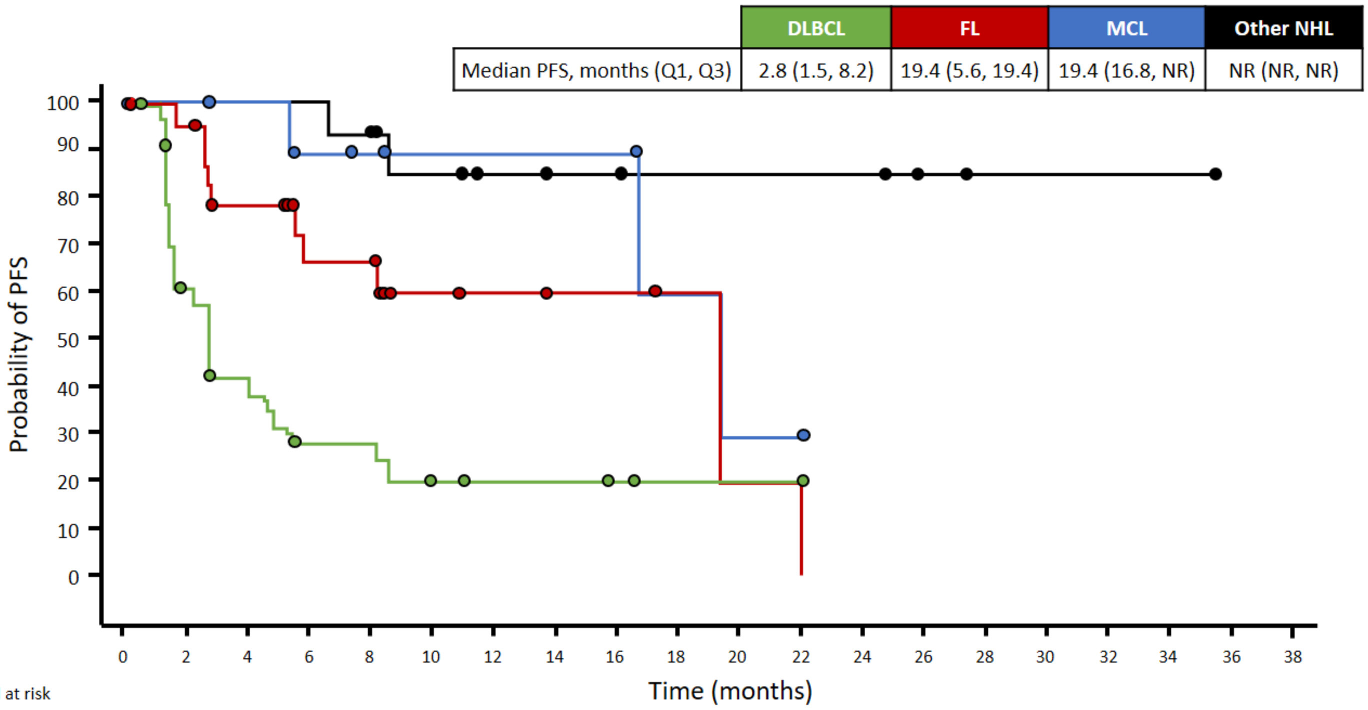

N at risk

	0	2	4	6	8	10	12	14	16	18	20	22	24	26	28	30	32	34	36
DLBCL	39	19	12	7	7	5	3	3	2	1	1	1	0						
FL	26	23	17	11	11	6	5	4	4	3	1	0							
MCL	11	10	9	7	6	5	5	5	5	2	1	1	0						
Other NHL	15	15	14	14	13	10	8	7	7	6	6	6	6	4	2	2	2	2	0

Supplemental Figure 5. MRD assessment by ctDNA clone tracking in DLBCL patients receiving A) TIRA/IDELA and B) TIRA/ENTO.

A

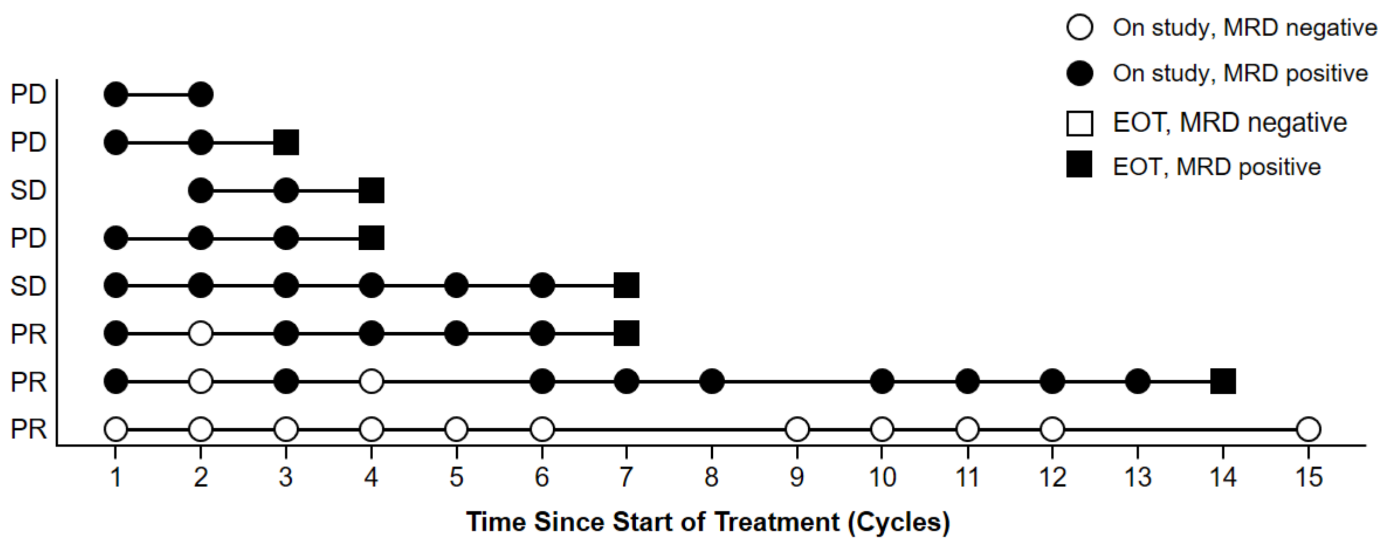

B

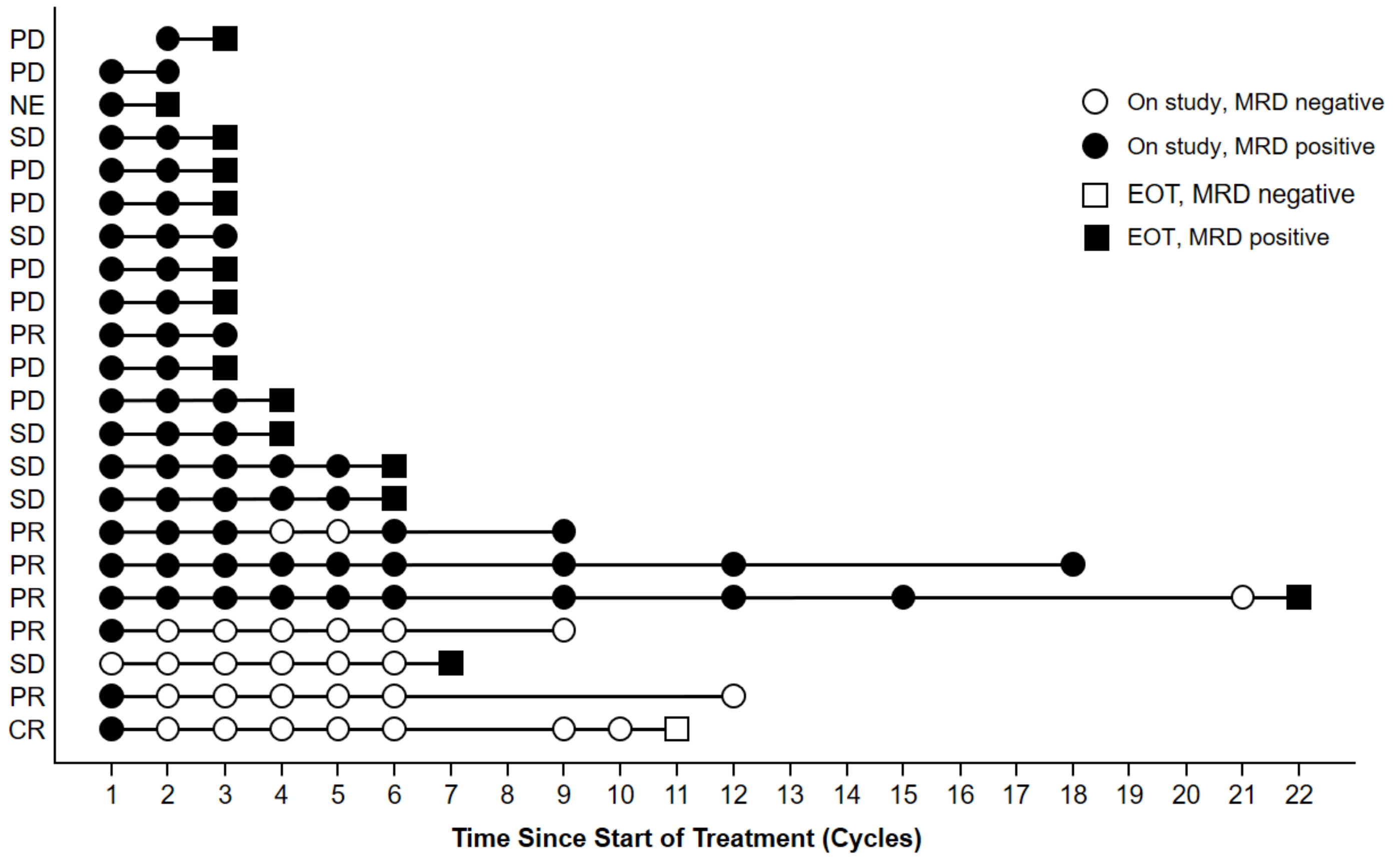

Cycle 1 sample was obtained before treatment and served as baseline.

CR, complete response; EOT, end of treatment; NE, not evaluable; PD, progressive disease; PR, partial response; SD, stable disease.
